# Development and validation of diagnostic models for immunoglobulin A nephropathy based on gut microbes

**DOI:** 10.3389/fcimb.2022.1059692

**Published:** 2022-12-08

**Authors:** Yijun Dong, Jiaojiao Chen, Yiding Zhang, Zhihui Wang, Jin Shang, Zhanzheng Zhao

**Affiliations:** ^1^ Department of Nephrology, the First Affiliated Hospital of Zhengzhou University, Zhengzhou, Henan, China; ^2^ School of Medicine, Zhengzhou University, Zhengzhou, Henan, China; ^3^ Nephrology Laboratory, the First Affiliated Hospital of Zhengzhou University, Zhengzhou, Henan, China; ^4^ Laboratory Animal Platform of Academy of Medical Sciences, Zhengzhou University, Zhengzhou, Henan, China

**Keywords:** glomerulonephritis, IgA, chronic kidney disease, gut microbiome, non‐invasive diagnostic tools, metabolic networks

## Abstract

**Background:**

Immunoglobulin A nephropathy (IgAN) is a highly prevalent glomerular disease. The diagnosis potential of the gut microbiome in IgAN has not been fully evaluated. Gut microbiota, serum metabolites, and clinical phenotype help to further deepen the understanding of IgAN.

**Patients and methods:**

Cohort studies were conducted in healthy controls (HC), patients of IgA nephropathy (IgAN) and non-IgA nephropathy (n_IgAN). We used 16S rRNA to measure bacterial flora and non-targeted analysis methods to measure metabolomics; we then compared the differences in the gut microbiota between each group. The random forest method was used to explore the non-invasive diagnostic value of the gut microbiome in IgAN. We also compared serum metabolites and analyzed their correlation with the gut microbiome.

**Results:**

The richness and diversity of gut microbiota were significantly different among IgAN, n_IgAN and HC patients. Using a random approach, we constructed the diagnosis model and analysed the differentiation between IgAN and n_IgAN based on gut microbiota. The area under the receiver operating characteristic curve for the diagnosis was 0.9899. The metabolic analysis showed that IgAN patients had significant metabolic differences compared with HCs. In IgAN, catechol, l-tryptophan, (1H-Indol-3-yl)-N-methylmethanamine, and pimelic acid were found to be enriched. In the correlation analysis, l-tryptophan, blood urea nitrogen and *Eubacterium coprostanoligenes* were positively correlated with each other.

**Conclusion:**

Our study demonstrated changes in the gut microbiota and established models for the non-invasive diagnosis of IgAN from HC and n_IgAN. We further demonstrated a close correlation between the gut flora, metabolites, and clinical phenotypes of IgAN. These findings provide further directions and clues in the study of the mechanism of IgAN.

## Introduction

Immunoglobulin A nephropathy (IgAN) is a well-known primary glomerular disease (Chou et al., 2012; O'Shaughnessy et al., 2017; Hu et al., 2020). It is characterized by recurrent attacks of hematuria and proteinuria. IgAN patients are prone to relapse, which brings great difficulties in disease treatment, resulting in a poor prognosis. The probability of IgAN eventually progressing to end-stage renal disease (ESRD) within 20-30 years of the first clinical presentation is approximately 40% (Lai et al., 2016). The pathology of IgAN is characterized by the deposition of immunoglobulin A (IgA) in the mesangium of the glomeruli, which can be detected by immunofluorescence staining (Wyatt and Julian, 2013).

The close relationship between IgAN and mucosal immunity has been established since 1968 when Berger and Hinglais (Berger and Hinglais, 1968) first reported IgAN. Further research confirmed that IgA deposits in the glomerular mesangium are mainly produced by mucosa-associated lymphoid tissue (MALT) (Béné and Faure, 1988; Kano et al., 2021). Many studies have recently reported that the incidence and severity of IgAN are closely related to the degree of intestinal mucosal injury (Gesualdo et al., 2021; Sallustio et al., 2021). Changes in the intestinal barrier can cause harmful effects, including endotoxin absorption through the intestinal barrier, overproduction of aberrantly glycosylated IgA1, which is the predominant IgA subtype (Suzuki et al., 2009; Makita et al., 2020), and IgA deposition in the mesangium of glomeruli (Suzuki et al., 2018). Several metabolites secreted by gut microbes, such as *Bacteroidetes*, help to maintain the stability of the immune system. Therefore, the correlation between the enteric-renal axis and IgAN is attracting increasing attention (Coppo, 2018; Zhong et al., 2020; Sallustio et al., 2021).

Although previous studies reported that the microbial community was substantially changed in patients with IgAN compared with healthy controls (HC) and membranous nephropathy (MN) patients (Dong et al., 2020), it is unclear whether IgAN can be diagnosed based on the gut microbiota and whether there are metabolic differences in IgAN. In this study, using a total of 441 fecal samples, the intestinal flora was compared between IgAN, non-IgAN (pathologically diagnosed non-IgA glomerular nephritis without secondary reasons, n_IgAN), and HC groups. Furthermore, the correlation between the intestinal flora, serum metabolites, and clinical phenotype was analyzed to deepen our understanding of the pathogenesis of IgAN.

## Materials and methods

### Inclusion and exclusion criteria

The diagnostic criteria for IgAN and n_IgAN (including MN, minimal change disease (MCD), focal segmental glomerular sclerosis (FSGS), and primary membranoproliferative glomerulonephritis (MPGN)) were based on renal biopsy findings. The exclusion criteria were as follows: 1) secondary IgAN; 2) patients who had received antibiotics, probiotics, prebiotics, laxatives, glucocorticoids or immunosuppressants within 3 months of sample collection; 3) individuals with a history of cholecystectomy, colectomy, confirmed hemorrhagic disease, or other intestinal diseases; 4) individuals with cancer, metabolic diseases, or severe infection; and 5) IgAN with other primary glomerular diseases.

**Figure 1:**
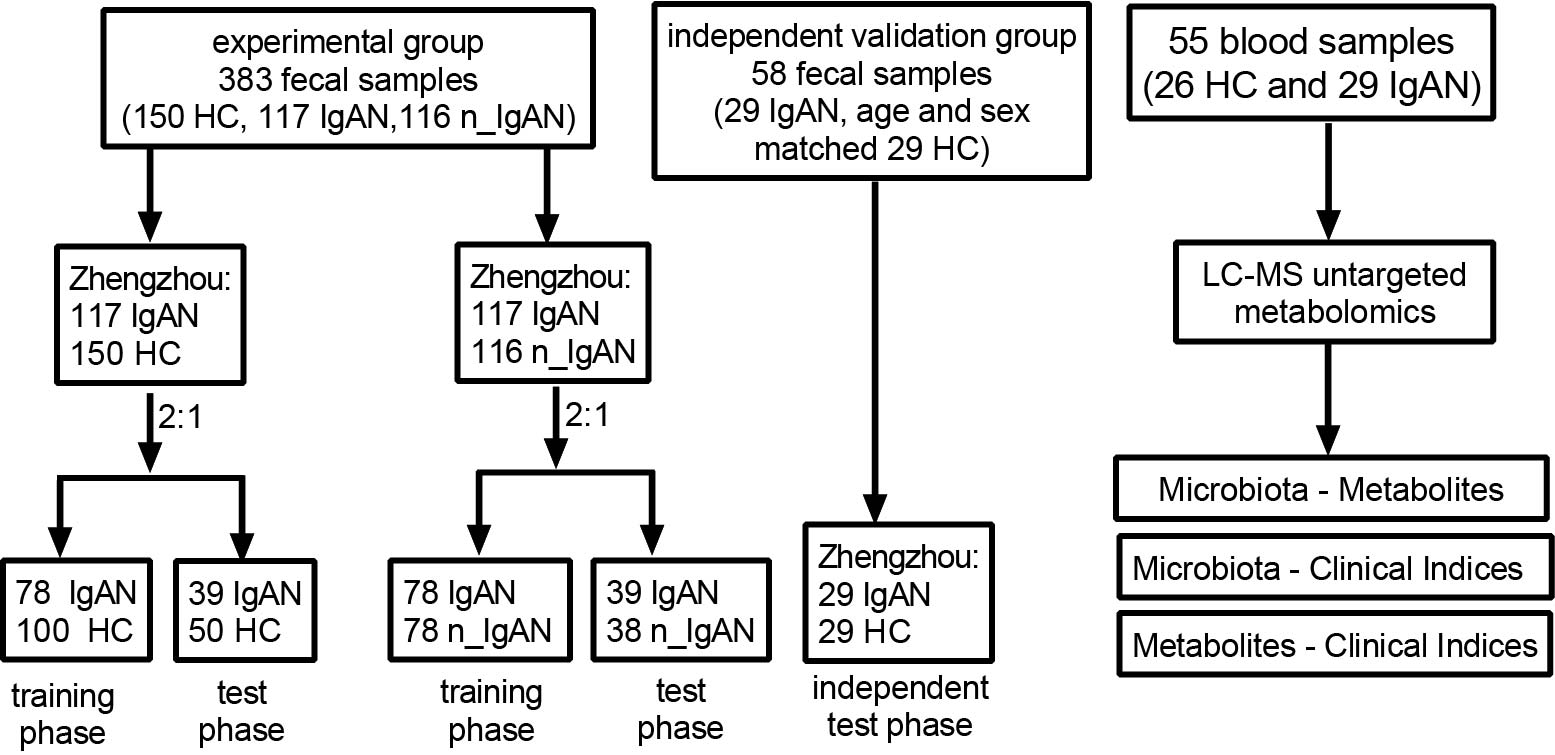
Flow chart of this study. Flow chart of disease diagnosis and differential diagnosis models based on fecal samples. In experimental group, 117 IgAN and 116 n_ IgAN fecal samples were collected from the biobank of the First Affiliated Hospital of Zhengzhou University from May 24, 2018, to February 1, 2021. One-hundred-and-fifty HCs fecal samples were collected from age- and sex- matched healthy volunteers. In independent validation group, 29 IgAN fecal samples were collected from the biobank of the First Affiliated Hospital of Zhengzhou University from February 15, 2021 to July 31, 2021. And another 29 HCs fecal samples were collected from age- and sex- matched healthy volunteers. A total of 150 HCs and 117 IgAN fecal samples were randomly divided into the training phase (100 HCs and 78 IgAN patients) and test phase (50 HCs and 39 IgAN patients) at a ratio of 2:1. At a ratio of 2:1, 117 IgAN and 116 n_IgAN were randomly divided into the training phase (78 IgAN patients and 78 n_IgAN patients) and test phase (39 IgAN patients and 38 n_IgAN patients). Flow chart of analysis of association of microbiota, metabolites and clinical indicators through serum metabolomics studies. In the blood samples, 29 IgAN patients were from the independent validation group, while 26 HCs was sex- and age- matched healthy controls. HC, healthy controls; IgAN, immunoglobulin A nephropathy; n_IgAN, non-IgA nephropathy; LC-MS, Liquid Chromatography-Mass Spectrometry.

Our study included two major parts, the experimental study and the independent validation study. In the experimental study, we retrospectively collected 117 IgAN and 116 n_ IgAN fecal samples from the biobank of the First Affiliated Hospital of Zhengzhou University from May 24, 2018, to February 1, 2021. In addition, 150 HC fecal samples were collected from age- and sex- matched healthy volunteers. In the independent validation study, we retrospectively collected 29 IgAN fecal samples from the biobank of the First Affiliated Hospital of Zhengzhou University from February 15, 2021, to July 31, 2021. In addition, another 29 HC fecal samples were collected from age- and sex- matched healthy volunteers. Regarding the blood samples, 29 IgAN samples were from the independent validation group, while an additional 26 HC were matched by sex and age. The study flow chart is shown in **Figure 1**.

### Ethical approval

The study was approved by the Ethics Committee of the First Affiliated Hospital of Zhengzhou University (2022-KY-0572-001). All participants signed written informed consents before enrollment.

### Sample collection and DNA extraction, 16S rRNA amplification, and gene sequencing

Fresh fecal samples were collected from the enrolled individuals in the morning. After collection, the samples were immediately frozen and stored at −80°C before analysis. The fecal sample was added to a 2 ml screwcap vial containing 1 g glass beads (0.1 mm BioSpec Products, Inc., USA) and was suspended in 790 µl sterile lysis buffer (4 M guanidine thiocyanate; 10% N-lauroyl sarcosine; 5% N-lauroyl sarcosine and 0.1 M phosphate buffer [pH 8.0]). The sample was subjected to bead beating for 10 min at maximum speed prior to incubation at 70°C for 60 min. Microbial DNA was extracted using the E.Z.N.A.^®^ Stool DNA Kit (Omega Bio-tek, Inc., GA, USA).

The primers F1 and R2 (5’- CCTACGGGNGGCWGCAG -3’ and 5’-GACTACHVGGGTATCTAATCC-3’) corresponding to positions 341 to 805 in the *Escherichia coli* 16S rRNA gene were used to amplify the V3~V4 region in each fecal sample by PCR. PCR reactions were run in a EasyCycler 96 PCR system (Analytik Jena Corp., AG) using the following program: 3 min of denaturation at 95°C followed by 21 cycles of 0.5 min at 94°C (denaturation), 0.5 min for annealing at 58°C, and 0.5 min at 72°C (elongation), with a final extension at 72°C for 5 min. The products from different samples were indexed and mixed at equal ratios for sequencing by Shanghai Mobio Biomedical Technology Co. Ltd. using the Miseq platform (Illumina Inc., USA), according to the manufacturer’s instructions.

### Bioinformatics and statistical analysis

Operational taxonomic units (OTUs) were classified with 97% similarity. Usearch (version 11 http://drive5.com/uparse/) was used to cluster the sequences. The Ribosomal Database Project Classifier algorithm (release 11.1 http://rdp.cme.msu.edu/) was applied to analyze the taxonomy of each 16S rRNA sequence using the Silva database (release 119 http://www.arb-silva.de).

Alpha diversity was estimated from the OTUs analysis and was presented using the ACE index, chao index, Shannon index, and Simpson index, which were analyzed using the implemented method in the R package “vegan”. Bacterial taxonomic comparison at the phylum and genus levels was tested between two groups using the Wilcoxon rank sum test, and the nonparametric Kruskal-Wallis rank sum test was used among three groups. Both weighted and unweighted UniFrac were aculeated in QIIME (v1.9.1). In addition, principal coordinate analysis (PCoA) plots and partial least squares discriminant analysis (PLSDA) were generated by the QIIME pipeline to visualize the UniFrac dissimilarity. The Venn diagram was used to compare the differences in intestinal flora based on OTUs between the two groups. Linear discriminant analysis (LDA) effect size (LEfSe) was used to determine the characteristic microbiota and explain the differences between the patients and HCs (lefse 1.1, https://github.com/SegataLab/lefse). Different characterizations were performed with an LDA cutoff of 3.0 and P value < 0.05.

For the correlation analysis, Spearman’s rank test was performed. The two-tailed-test was used to evaluate continuous variables and the Chi-square test was used to compare categorical variables between the two groups. Statistical analyses were performed using SPSS version 22.0 (IBM SPSS, Chicago, IL, USA). P-values were regarded to be significant at P < 0.05. Phylogenetic Investigation of Communities by Reconstruction of Unobserved States 2 (PICRUSt2) v2.4.1 (https://github.com/picrust/picrust2/wiki) was used to find significant different metabolic pathways and orthologous groups between the two groups compared with the Kyoto Encyclopedia of Genes and Genomes (KEGG) database (LDA scores > 3.0, P < 0.05).

### OTU selection and model construction

To evaluate the potential of gut microbial markers as non-invasive diagnostic and differential diagnostic tool for IgAN, we constructed random forest classifier models. In the diagnostic model (117 IgAN and 150 HC), we used the Wilcoxon test to identify significance (P < 0.05). Five-fold cross-validation was performed on a random forest model (R 3.4.1, random forest 4.6–12 package), and four OTUs were selected as the optimal marker set of IgAN by a five-fold cross-validation of the random forest model. We then obtained the cross-validation error curve; the point with the lowest cross-validation error was seen as the cut-off point, which was determined *via* the minimum error plus the standard deviation (SD). Then we chose all sets of OTU markers with the error less than the cut-off value and chose the set with the smallest number of OTUs as the optimal set. The probability of disease (POD) index was defined as the ratio between the number of randomly generated decision trees that predicted the sample as IgAN and that of HCs. The identified optimal set of OTUs was finally used for calculation of the POD index in both the training, testing, and independent phase. The receiver operating characteristic (ROC) curve was generated (R 3.3.0, pROC package) for evaluation of the constructed models; the area under the ROC curve (AUC), generated in R (http://www.R-project.org/), was used to designate the ROC effect. The differential diagnostic model (117 IgAN and 116 n_IgAN) was generated in the same way.

### Untargeted metabolomic assays

Fifty-five samples (26 HC and 29 IgAN) were collected for non-targeted metabolomics methods. All serum samples, collected in the morning after fasting, were put into 5 ml tubes (BD Vacutainer^®^ SST II Advance tube). The samples were centrifuged at 3,000 rpm for 10 min and stored at -80°C before analysis. The experimental sample was thawed at 4°C, vortexed for 1 min, and mixed evenly, before accurately transferring the sample into a 2 mL centrifuge tube. We then added 400 µL methanol (stored at -20°C) and the mixture was vortexed for 1 min. After centrifuging (Hunan Xiangyi Experiment Equipme Co., H1850-R) for 10 min at 12,000 rpm and 4°C. For concentration, the supernatant was transferred into a new 2 mL centrifuge tube. One-hundred-and-fifty microliters of 2-chloro-l-phenylalanine (4 ppm) solution, prepared with 80% methanol water (stored at 4°C), was used to redissolve the sample; the supernatant was filtered through a 0.22 μm membrane (Tianjin Jinteng Experiment Equipment Co., 0.22 μm PTFE) and transferred into the detection bottle for liquid chromatography-mass spectrometry (LC-MS). The LC analysis was performed on a ACQUITY UPLC System (Waters, Milford, MA, USA). Chromatography was carried out with an ACQUITY UPLC ^®^ HSS T3 (150 × 2.1 mm, 1.8 µm) (Waters, Milford, MA, USA), maintained at 40°C. MS detection of metabolites was performed on Q Exactive (Thermo Fisher Scientific, USA) with electrospray ion source. Simultaneous MS1 and MS/MS (full MS-ddMS2 mode, data-dependent MS/MS) acquisition was performed.

The raw data were firstly converted to mzXML format by MSConvert in ProteoWizard software package (v3.0.8789) and was processed using XCMS for feature detection, retention time correction, and alignment. The metabolites were identified by accuracy mass (< 30 ppm) and MS/MS data, which were matched with the Human Metabolome Database (HMDB; (http://www.hmdb.ca), massbank (http://www.massbank.jp/), LipidMaps (http://www.lipidmaps.org), mzclound (https://www.mzcloud.org) and KEGG (http://www.genome.jp/kegg/). In order to correct systematic bias, the robust quality control locally estimated scatterplot smoothing (LOESS) signal correction (QC-RLSC) was applied to normalize our data. Then, only ion peaks with a relative SD < 30% in QC-RLSC were kept, ensure proper metabolite identification. The ropls software was used for data analyses and modelling. Data were mean centered using scaling. Models were built on principal component analysis (PCA), orthogonal partial least-square discriminant analysis (PLS-DA), and partial least-square discriminant analysis (OPLS-DA). A score plot could visualize the metabolic profiles, and each point represents a sample. The metabolites, influence clustering of the samples, are shown with loading plot and S-plot. Permutation tests could be used to test the model efficacy for over fitting. The descriptive function of the models was determined by R2X (cumulative) (perfect model: R2X (cum) = 1) and R2Y (cumulative) (perfect model: R2Y (cum) = 1) values, while their prediction performance was measured by Q2 (cumulative) (perfect model: Q2 (cum) = 1) and a permutation test. The permuted model should not be able to predict classes: R2 and Q2 values at the Y-axis intercept must be lower than those of Q2 and the R2 of the non-permuted model. OPLS-DA allowed the discrimination of metabolites using the variable importance on projection (VIP). The P value, VIP produced by OPLS-DA, and fold change was applied to discover the contributable-variable for classification. Finally, those with P values < 0.05 and VIP values > 1 were considered to be statistically significant metabolites.

### Statistical analysis

Statistical analyses were conducted using SPSS and R software. For demographics and laboratory data, quantitative data were presented either as medians with interquartile ranges (IQR) or means ± SD, whereas categorical variables were presented as percentages and were compared using the Chi-square test or Fisher’s exact test. Continuous variables were assessed using an un-paired t-test or a nonparametric test. Biological databases, such as the HMDB and KEGG, were used to identify biomarkers, metabolic pathways and gene function.

## Results

### Details of the included samples

After strict screening, a total of 467 patients (205 HCs, 146 IgAN and 116 n_IgAN) were included in this study (**Figure 1**). At first step, we retrospectively collected 117 IgAN fecal samples from the biobank of the First Affiliated Hospital of Zhengzhou University from May 24, 2018, to February 1, 2021. In addition, 150 HCs fecal samples were collected from age- and sex- matched healthy volunteers. A total of 150 HCs and 117 IgAN samples were randomly divided into the training phase (100 HCs and 78 IgAN) and test phase (50 HCs, 39 IgAN) at a ratio of 2:1. Secondly, in order to check the validity of the diagnostic model, we retrospectively collected fecal samples of another cohort from February 15, 2021, to July 31, 2021. This cohort included 29 IgAN fecal samples. In addition, another 29 HCs fecal samples were collected from age- and sex- matched healthy volunteers.

To distinguish IgAN patients from other glomerular diseases, we retrospectively collected 116 n_IgAN fecal samples from the biobank of the First Affiliated Hospital of Zhengzhou University from April 27, 2019, to February 1, 2021. The 116 n_IgAN fecal samples included 76 MN, 34 MCD, five FSGS and one MPGN. The two groups, 117 IgAN and 116 n_ IgAN patients, were randomly divided into training phase (78 IgAN and 78 n_ IgAN) and test phase (39 IgAN and 38 n_ IgAN) at a ratio of 2:1. In n_IgAN group, there were 54 MN, 21 MCD, 2 FSGS and 1 MPGN patients in training phase and 22 MN, 13 MCD, 3 FSGS patients in test phase.

Bloods of the 29 IgAN patients collected from February 15, 2021, to July 31, 2021 from the biobank of the First Affiliated Hospital of Zhengzhou University were collected for Untargeted Metabolomics Analysis, while an additional 26 HCs patients were matched by sex and age.

### The richness and diversity of the gut microbiota were significantly different among the three groups

**Figure 2:**
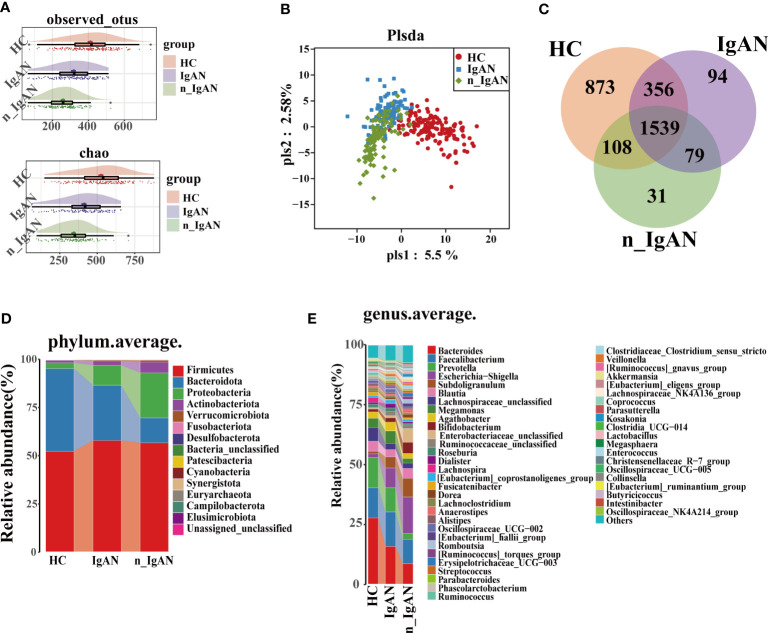
Gut microbiome composition differences in HC, IgAN and n_IgAN groups. **(A)** Observed OTUs and chao index were decreased in IgAN and n_IgAN groups. **(B)** PLSDA graph of gut microbiota composition. **(C)** A Venn diagram based on distribution of intestinal microbe OTUs. At the phylum **(D)** and genus **(E)** levels, there were significant differences in the content of gut microbiota between three groups. PLSDA, partial least squares discriminant analysis.

The abundance, distribution, and diversity of the microbial species in IgAN and n_IgAN patients were lower than those of HCs. The OTUs and alpha diversity of intestinal flora was significantly different (**Figure 2A**, **Figure S1A**, **Table S1**). Results of PLSDA are shown in **Figure 2B**. As shown in the Venn diagram (**Figure 2C**), 1539 OTUs were shared by the three groups. At phylum and genus levels (**Figure 2D, E**, **Figure S1B**), there were significant differences in the content of the gut microbiota between the three groups. In addition, we investigated the KEGG pathways at each of the different levels (**Figure S1C, D**).

### The intestinal microbial model has strong diagnostic potential for IgAN

**Figure 3:**
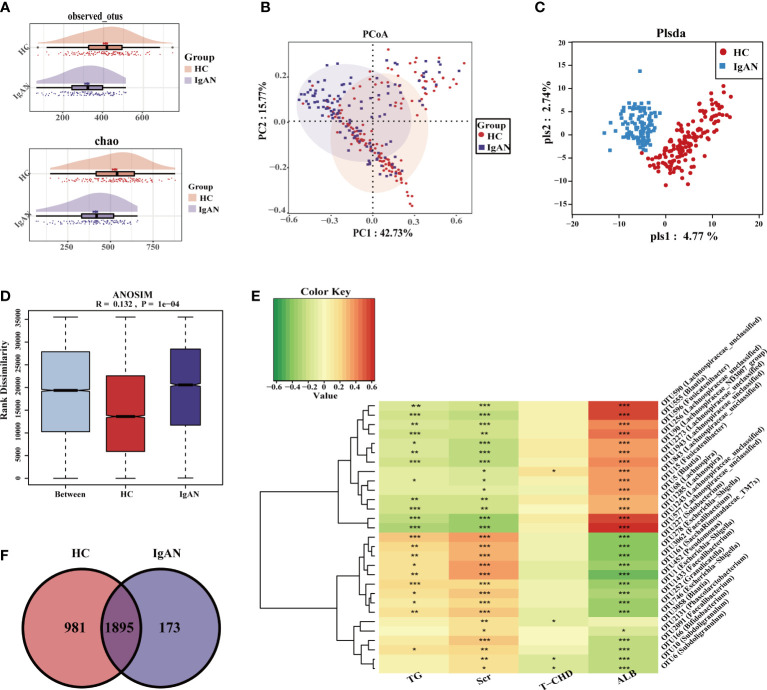
Gut microbiome composition differences at OUT level in HC and IgAN. **(A)** Observed OTUs and chao index were decreased in IgAN. **(B)** Two-dimensional PCoA based on weighted UniFrac distance between HC and IgAN. **(C)** PLSDA graph of the composition of gut microbiota in HC and IgAN. **(D)** ANOSIM results indicated that whether the difference between groups and within groups were statistically different. P < 0.05 indicates that the statistics are significant. **(E)** A Venn diagram based on the distribution of intestinal microbe OTUs in two groups. **(F)** Spearman correlations between different OTUs and clinical indicators. Red represented a positive relationship, and green represented a negative relationship. The darker the color, the stronger the correlation. The correlation with significant difference is indicated by an asterisk(*P < 0.05; **P < 0.01; ***P < 0.001). OTUs, Operational Taxonomic Units; PCoA, Principal Coordinates Analysis; PLSDA, partial least squares discriminant analysis; ANOSIM, analysis of similarity; TG, triglycerides; Scr, Serum creatinine; T-CHO, total cholesterol; ALB, serum albumin; HC, healthy controls; IgAN, immunoglobulin A nephropathy.

Serum creatinine (Scr) in IgAN patients was higher than in HCs, while serum albumin (ALB) was lower than in HCs (**Table 1**). Compared with HCs, the OTUs and alpha diversity of the intestinal flora was significantly decreased in IgAN patients (**Figure 3A**, **Figure S2A**, **Table S1**). PCA (**Figure S2B**), PCoA (**Figure 3B**), and PLSDA (**Figure 3C**) showed significant deviation between IgAN patients and HCs (P < 0.05). The analysis of the similarities (ANOSIM) illustrated trustworthy grouping (**Figure 3D**, R=0.132, P = 0.0001). As shown in the Venn diagram (**Figure 3E**), there were 173 unique OTUs in IgAN. Spearman correlation analysis was conducted between the different OTUs and clinical indicators (**Figure 3F**). In IgAN, OTU452 (*Pseudomonas*), OTU161 (*Saccharimonadaceae-TM7x*), OTU227 (*Solobacterium*), OTU3062 (*Faecalibacterium*) and OTU278 (*Escherichia-Shigella*) were negatively correlated with ALB, and positively correlated with Scr. Furthermore, OTU2273 (*Lachnospiraceae-unclassified*) and OTU555 (*Blautia*) were positively correlated with ALB, but negatively correlated with Scr.

**Table 1:** Baseline characteristics of HC and IgAN.

characteristic	Overall	HC	IgAN	p
	n=267	n=150	n=117	
**Male, n (%)**	146 (54.7)	80(53.3)	66(56.4)	0.706
**Age, year, median [IQR]**	40.00 [37.00, 50.00]	40.00 [38.00, 50.00]	42.00 [30.00, 52.00]	0.135
**HB, g/L, mean ± SD**	136.95 ± 20.34	145.19 ± 15.81	126.38 ± 20.65	**<0.001**
**BUN, mmol/L, median [IQR]**	5.11 [4.20, 6.48]	4.62 [3.88, 5.40]	6.30 [4.90, 8.50]	**<0.001**
**Scr, umol/L, median [IQR]**	75.00 [63.00, 92.00]	69.00 [59.00, 77.75]	96.00 [75.00, 128.00]	**<0.001**
**UA, umol/L, median [IQR]**	313.00 [257.50, 381.00]	290.50 [244.50, 354.00]	350.00 [285.00, 426.00]	**<0.001**
**ALB, g/L, median [IQR]**	45.80 [39.70, 48.80]	48.45 [46.90, 50.08]	38.60 [35.10, 41.40]	**<0.001**
**T-CHO, mmol/L, median [IQR]**	4.61 [3.99, 5.22]	4.69 [4.19, 5.16]	4.42 [3.58, 5.26]	**0.010**
**TG, mmol/L, median [IQR]**	1.27 [0.90, 1.80]	1.08 [0.88, 1.40]	1.63 [1.00, 2.42]	**<0.001**
**HDL, mmol/L, median [IQR]**	1.28 [1.05, 1.54]	1.43 [1.24, 1.71]	1.05 [0.92, 1.24]	**<0.001**
**LDL, mmol/L, median [IQR]**	2.82 [2.36, 3.30]	2.88 [2.49, 3.30]	2.64 [2.17, 3.31]	0.104
**eGFR, ml/min/m^2^, median [IQR]**	99.66 [80.26, 110.14]	105.01 [97.54, 111.70]	72.92 [50.73, 98.71]	**<0.001**
**24hTP, g, median [IQR]**	——	——	1.7[0.81,3.09]	——

SD, standard deviation; IQR, the interquartile range; HC, healthy controls; IgAN, immunoglobulin A nephropathy. HB, hemoglobin; BUN, blood urea nitrogen; Scr, Serum creatinine; UA, uric acid; ALB, serum albumin; T-CHO, total cholesterol; TG, triglycerides; HDL, high density lipoprotein; LDL, low density lipoprotein; eGFR, estimated glomerular filtration rate; 24hTP, 24-hour urinary total protein levels. Bold data in P values indicated statistically significant differences between two groups.

The normal quantitative data was expressed as mean ± SD, the non-normal quantitative data was expressed as median [IQR], and the qualitative data was expressed as numerical (percentage).

**Figure 4:**
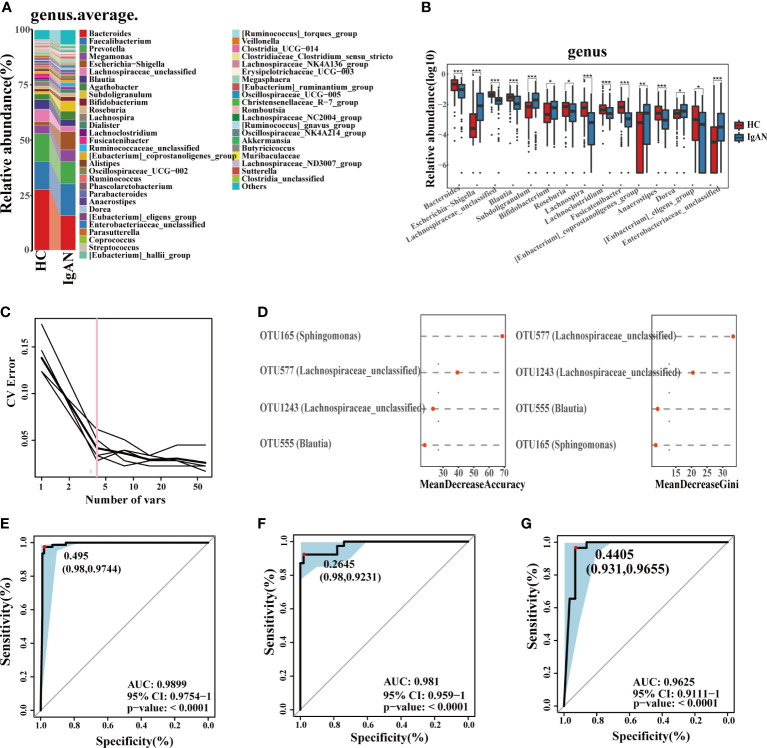
Phylogenetic profiles and diagnostic potential of gut microbes. **(A)** Comparison of microbial composition on genus level. **(B)** Comparison of differential microorganisms on genus level. Log_10_ abundance of differential microorganisms on genus level. Statistically significant differences according to the Wilcoxon rank-sum test are marked with asterisks (*P < 0.05; **P < 0.01; ***P < 0.001). **(C, D)** Four microbial OTUs were finally determined as the optimal marker set to diagnose IgAN by the random forest model between HC and IgAN. The POD values of the training phase **(E)**, test phase **(F)** and independent test phase **(G)**, obtained AUC values of 0.9899, 0.981 and 0.9625, respectively. CV, coefficient of variation; OTUs, Operational Taxonomic Units; POD, probability of disease; CI, confidence interval; AUC, area under the curve.

At the phylum level (**Figure S2C, D**), the abundance of *Proteobacteria*, *Actinobacteriota* and *Patescibacteria* in IgAN was higher than HCs (P < 0.05). At the genus level (**Figure 4A, B**), bacteria were enriched in IgAN, including *Escherichia-Shigella*, *Subdoligranulum*, *Bifidobacterium*, *Dorea*, etc. According to the LDA histogram (**Figure S2E**), 11 genera were significantly enriched in IgAN, while 13 genera were significantly enriched in HCs. A random forest classifier was developed to distinguish IgAN from HCs. After five-fold cross-validation, four microbial OTUs were finally determined as the optimal marker set (**Figure 4C, D**). The calculated POD indices of IgAN in the training phase (**Figure S2F**), test phase (**Figure S2G**), and independent test phase (**Figure S2H**) were significantly higher than in HCs (P < 0.05). In the train phase, the AUC between IgAN and HC reached a statistically significant result at 0.9899 (P < 0.0001, **Figure 4E**). The AUC in the test phase and independent test phase was 0.981 and 0.9625, respectively (**Figure 4F, G**). These results indicated that the classifier had strong diagnostic potential for IgAN.

### Correlation of microbiota-metabolites-phenotypes in IgAN

**Figure 5:**
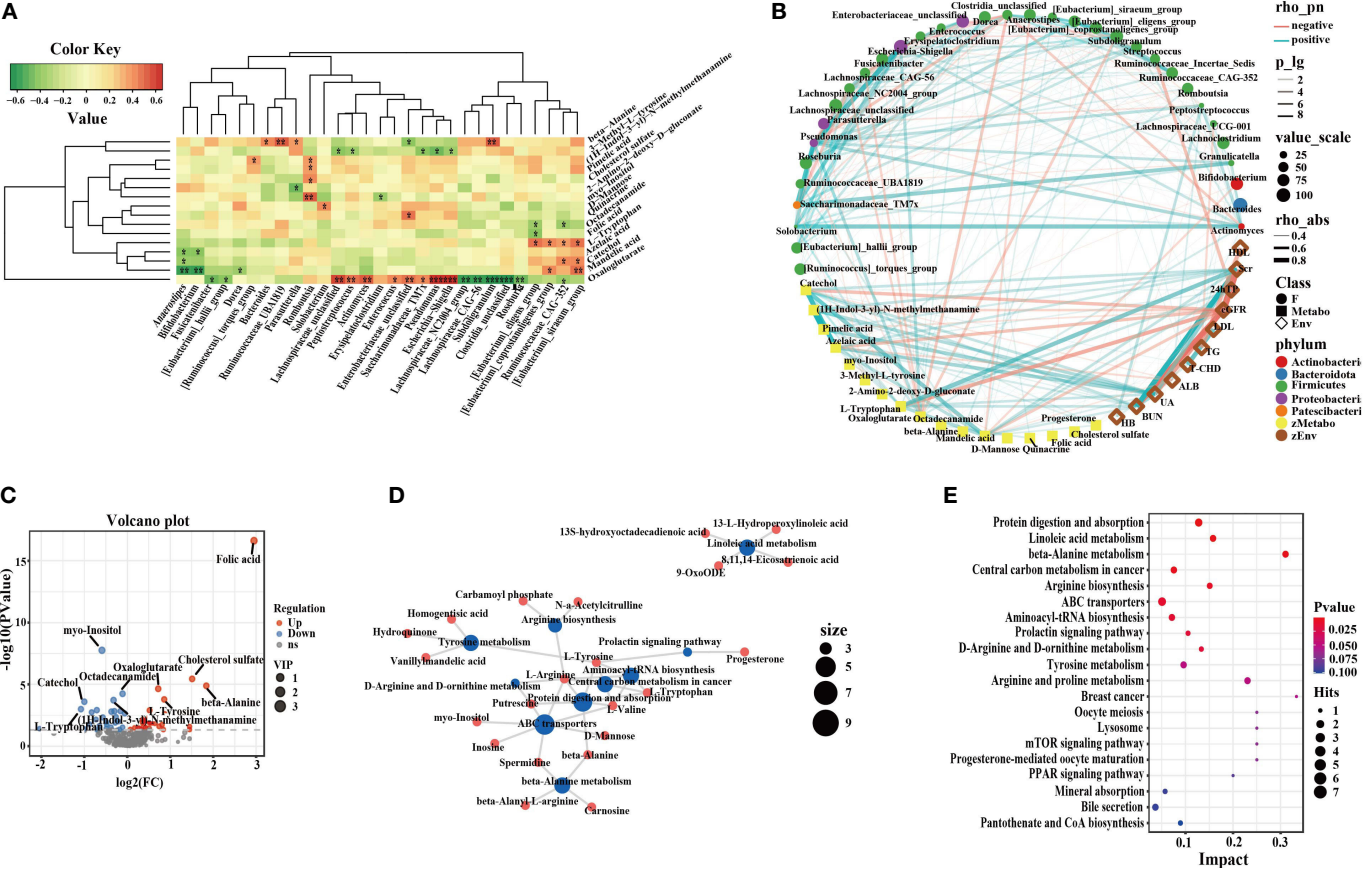
Correlation of intestinal microflora, metabolites and clinical phenotypes and differential metabolic pathways. **(A)** The correlation analysis of differential flora and metabolites in IgAN (*P < 0.05; **P < 0.01; ***P < 0.001). **(B)** Network graph of intestinal microflora, differential metabolites and clinical indices. **(C)** Volcano plots of differential metabolites in HC. The red dots represented up-regulated differentially expressed metabolites, the blue dots represented down-regulated differentially expressed metabolites, and the gray dots represented metabolites that were detected but did not meet the filtering parameter screening. **(D)** Network graph of differential metabolic pathway influencing factors between IgAN patients and HCs. The blue points represented pathways, and the red points represented metabolites, and the size of the points represented the number of points connected to them. **(E)** Bubble graph of differential metabolic pathway influencing factors. Each point represented a metabolic pathway. The abscissa was the impact value enriched in different metabolic pathways, and the ordinate was the enriched pathways. And the dots represented the number of corresponding metabolic molecules on the pathway. OPLS-DA, Orthogonal Partial Least Squares-Discriminant Analysis; PPAR, peroxisome proliferation-activated receptor; mTOR, mammalian target of rapamycin; HB, hemoglobin; BUN, blood urea nitrogen; Scr, Serum creatinine; UA, uric acid; ALB, serum albumin; T-CHO, total cholesterol; TG, triglycerides; HDL, high density lipoprotein; LDL, low density lipoprotein; eGFR, estimated glomerular filtration rate; 24hTP, 24-hour urinary total protein levels; HC, healthy controls; IgAN, immunoglobulin A nephropathy.

We used untargeted metabolomics to analyze the differential metabolites in 26 HCs and 29 IgAN patients. The baseline data is shown in **Table 2**. We further performed association and cluster analysis on the differential metabolites and clinical indicators (**Figure S3A, B**, **Figure 5A, B**).

**Table 2:** Comparison of baseline characteristics of IgAN and HC groups in untargeted Metabolomics analysis.

characteristic	Overall	HC	IgAN	p
	n=55	n=26	n=29	
**Male, n (%)**	25 (45.5)	13 (50.0)	12 (41.4)	0.712
**Age, year, median [IQR]**	37.00 [31.50, 48.50]	36.00 [32.00, 54.00]	40.00 [31.00, 47.00]	0.135
**HB, g/L, median [IQR]**	138.00 [121.50, 153.00]	149.00 [131.50, 157.75]	133.00 [114.00, 141.00]	**<0.001**
**BUN, mmol/L, median [IQR]**	5.40 [4.19, 7.30]	4.21 [3.80, 6.12]	5.80 [4.60, 9.00]	**<0.001**
**Scr, umol/L, median [IQR]**	74.00 [64.50, 98.41]	71.00 [62.50, 82.25]	88.40 [71.00, 131.00]	**<0.001**
**UA, umol/L, mean ± SD**	343.35 ± 104.59	311.67 ± 91.18	371.76 ± 109.12	**<0.001**
**ALB, g/L, mean ± SD**	43.18 ± 6.31	47.39 ± 3.65	39.41 ± 5.81	**<0.001**
**T-CHO, mmol/L, mean ± SD**	4.51 ± 0.71	4.38 ± 0.81	4.62 ± 0.59	**0.010**
**TG, mmol/L, median [IQR]**	1.49 [1.06, 1.90]	1.31 [0.98, 1.73]	1.58 [1.30, 2.18]	**<0.001**
**HDL, mmol/L, mean ± SD**	1.24 ± 0.30	1.29 ± 0.25	1.20 ± 0.34	**<0.001**
**LDL, mmol/L, mean ± SD**	2.71 ± 0.68	2.62 ± 0.74	2.79 ± 0.62	0.104
**eGFR, ml/min/m^2^, median [IQR]**	92.64 [76.78, 108.74]	104.87 [90.72, 115.94]	83.97 [57.85, 97.81]	**<0.001**
**24hTP, g, median [IQR]**	——	——	1.46 [0.54, 2.88]	——

The normal quantitative data was expressed as mean ± SD, the non-normal quantitative data was expressed as median [IQR], and the qualitative data was expressed as numerical (percentage). SD, standard deviation; IQR, the interquartile range; HC, healthy controls; IgAN, immunoglobulin A nephropathy. HB, hemoglobin; BUN, blood urea nitrogen; Scr, Serum creatinine; UA, uric acid; ALB, serum albumin; T-CHO, total cholesterol; TG, triglycerides; HDL, high density lipoprotein; LDL, low density lipoprotein; eGFR, estimated glomerular filtration rate; 24hTP, 24-hour urinary total protein levels. Bold data in P values indicated statistically significant differences between two groups.

In IgAN, enriched catechol, azelaic acid, mandelic acid, and l-tryptophan were positively correlated with Scr, blood urea nitrogen (BUN), uric acid (UA), and 24 total urinary protein (24hTP), but negatively correlated with the estimated glomerular filtration rate (eGFR) and ALB. Azelaic acid and mandelic acid were negatively correlated with *Bifidobacterium*. *Bifidobacterium* was positively correlated with ALB. There were positive correlations between the serum metabolite catechol, the enteric *Eubacterium-siraeum* and total cholesterol (T-CHO). L-tryptophan, BUN, and *Eubacterium-coprostanoligenes* were positively correlated with each other. Furthermore, UA, (1H-Indol-3-yl)-N-methylmethanamine, and *Romboutsia* were positively correlated with each other. UA, pimelic acid and *Romboutsia* were positively correlated with each other. Myo-inositol was negatively correlated with triglycerides (TG), while octadecanamide was positively correlated with high density lipoprotein (HDL). We performed OPLS-DA on polar compounds. Peripheral blood metabolomics of IgAN patients and HCs had significantly different distribution trends (**Figure S3C, D**). There were 26 metabolites with up-regulated expression in IgAN, including myo-Inositol, (1H-Indol-3-yl)-N-methylmethanamine, catechol, pimelic acid, etc., and 32 metabolites with down-regulated expression, including folic acid, octadecanamide, l-tyrosine, etc. The volcano plots could clearly characterize the distribution of differential metabolites between the two groups (**Figure 5C**). Differential metabolites are listed in **Figure S3E, F**. The MetaboAnalyst software was used to analyze key metabolic pathways, and to evaluate the importance of each metabolic pathway in IgAN. We screened out the potential target metabolic pathways with a pathway impact > 0.1 (**Figure 5D, E**, **Table S3**). IgAN was mainly involved in eight metabolic pathways.

### The gut microbiome varies between IgAN and n_IgAN patients

**Figure 6:**
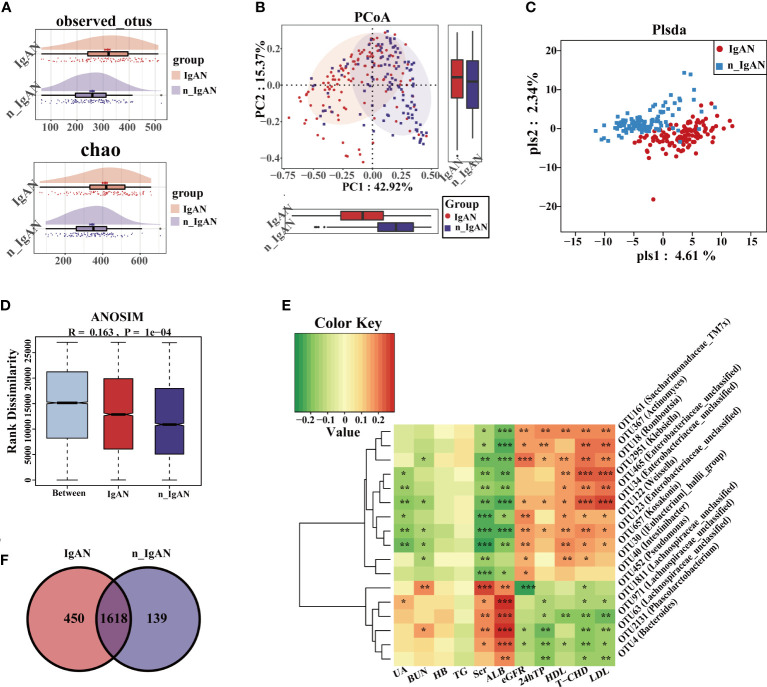
Differences in composition of gut microbiome at OUT level in IgAN and n_IgAN. **(A)** Observed OTUs and chao index were increased in IgAN. **(B)** Two-dimensional PCoA based on weighted UniFrac distance. **(C)** PLSDA graph of the composition of gut microbiota. **(D)** ANOSIM graph showed differences between groups is greater than differences within groups. **(E)** A Venn diagram based on the distribution of intestinal microbe OTUs in two groups. **(F)** Spearman correlations between different OTUs and clinical indicators. The correlation with significant difference is indicated by an asterisk (*P < 0.05; **P < 0.01; ***P < 0.001). PCoA, Principal Coordinates Analysis; PLSDA, partial least squares discriminant analysis; ANOSIM, analysis of similarity; HB, hemoglobin; BUN, blood urea nitrogen; Scr, Serum creatinine; UA, uric acid; ALB, serum albumin; T-CHO, total cholesterol; TG, triglycerides; HDL, high density lipoprotein; LDL, low density lipoprotein; eGFR, estimated glomerular filtration rate; 24hTP, 24-hour urinary total protein levels; HC, healthy controls; n_IgAN, non-IgA nephropathy.

**Figure 7:**
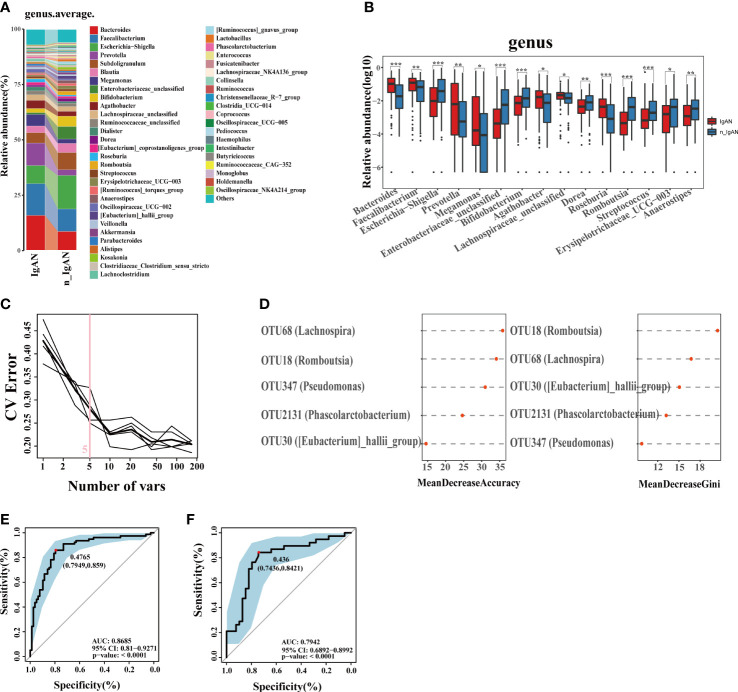
Phylogenetic profiles and differential diagnostic potential of gut microbes. **(A)** Comparison of microbial composition on genus level. **(B)** Comparison of differential microorganisms on genus level. Log_10_ abundance of differential microorganisms on genus level (*P < 0.05; **P < 0.01; ***P < 0.001). **(C, D)** Five microbial OTUs were finally determined as the optimal marker set for differential diagnose IgAN by the random forest model between IgAN and n_IgAN. The POD values of the training phase **(E)** and test phase **(F)** obtained AUC values of 0.8853 and 0.8448 respectively. CV, coefficient of variation; POD, probability of disease; CI, confidence interval; AUC, area under the curve.

The observed OTUs and alpha diversity of the intestinal flora were significantly increased in IgAN compared with n_IgAN patients (**Figure 6A**, **S4A**, **Table S1**). Based on the distribution of OTUs, PCA (**Figure S4B**), PCoA (**Figure 6B**), and PLSDA (**Figure 6C**) showed significant deviation between IgAN and n_IgAN patients (P < 0.05). The ANOSIM graph confirmed our reliable grouping (**Figure 6D**, R = 0.163, P = 0.0001). According to the Venn diagram (**Figure 6E**), 1618 OTUs were shared by IgAN and n_IgAN. Spearman correlation analysis of the different OTUs and clinical indicators was conducted (**Figure 6F**). OTU63 (*Lachnospiraceae-unclassified*) and OTU452 (*Pseudomonas*) were negatively correlated with eGFR and 24hTP, and positively correlated with ALB, BUN, and Scr. OTU18 (*Romboutsia*) was positively correlated with eGFR, but negatively correlated with ALB, BUN, and Scr. We further compared the biological composition and changes of intestinal microbes in IgAN and n_IgAN. At the phylum level, the abundance of *Proteobacteria*, *Actinobacteriota* and *Patescibacteria* in n_IgAN was enriched compared to IgAN (P < 0.05) (**Figure S4C, D**). At the genus level (**Figure 7A, B**), we compared the relative abundances of the top 15 different genera with the highest content among groups; we found a higher proportion of the following genera with in IgAN: *Bacteroides*, *Faecalibacterium*, *Prevotella*, *Megamonas*, *Agathobacter*, *Lachnospiraceae-unclassified*, *Roseburia* and *Anaerostipes*. According to the LDA value distribution histogram (**Figure S4E**), 24 genera were significantly enriched in n_IgAN, while 21 genera were significantly enriched in IgAN.

### The gut microbiome can non-invasively differentiate IgAN from GN

At a ratio of 2:1, 117 IgAN and 116 n_IgAN patients were randomly divided into the training phase (78 IgAN and 78 n_IgAN) and test phase (39 IgAN and 38 n_IgAN). A random forest classifier was developed. Five microbial OTUs were finally determined as the optimal marker set (**Figure 7C, D**). In the training phase (**Figure S4F**) and test phase (**Figure S4G**), the POD of n_IgAN increased significantly compared with that in IgAN (P < 0.05). In the training phase, the AUC reached a statistically significant result (AUC = 0.8685, P<0.0001, **Figure 7E**). The AUC in the test phase was 0.7942 (**Figure 7F**).

## Discussion and conclusion

A total of 441 stool samples were included to explore the non-invasive diagnostic and differential diagnostic value of human gut microbiome for IgAN. The proportion of n_IgAN spectrum was collected according to epidemiological statistics (Hu et al., 2020). Besides, 55 serum samples were collected to analyze the characteristics of serum metabolites and their correlation with gut microbiome. In this article, we established two models. One for the diagnosis of IgAN from HC, and the other for differential diagnosis of IgAN from other kinds of glomerulonephritis. Another feature of this study was the correlation analysis of serum metabolites, intestinal flora and clinical indicators, which laid a certain foundation for further study of mechanism.

Compared with HC, at the phylum level, the main difference was that the abundance of *Proteobacteria* and *Actinobacteria* increased significantly in IgAN and n_IgAN. These results were consistent with previous literature (Sugurmar et al., 2021). *Proteobacteria* includes many confirmed pathogenic bacteria or opportunistic pathogens, such as *Escherichia coli*, *Salmonella*, *Helicobacter pylori*, etc. And the increased abundance of *Proteobacteria* can be seen as a microbial marker of gut dysbiosis (Shin et al., 2015).

At the genus level, the relative abundance of *Bacteroidetes* in IgAN, a short-chain fatty acids (SCFAs)-producing bacteria (including *Bacteroidetes*, *Lachnospira*, *Blautia*, *Anaerostipes*, etc.), was lower than that of HCs. Metabolites secreted by different *Bacteroidetes* help to maintain the stability of the immune system. Unlike lipopolysaccharides (LPS), SCFAs exert beneficial effects on nourishing the colonic epithelium, regulating the intracellular potential of hydrogen (pH) (DeSoignie and Sellin, 1994), regulating blood pressure (Qu et al., 2022), and having anti-inflammation and anti-tumor effects (Tan et al., 2014; Vicentini et al., 2021),. Researchers have found that SCFAs-producing bacteria were significantly reduced in ESRD patients, with the progression to ESRD potentially related to loss of the renal-protective effect of SCFAs (Verkaik et al., 2010; Wong et al., 2014; Sabatino et al., 2017; Wang et al., 2019). Andrade-Oliveira et al. (Andrade-Oliveira et al., 2015) concluded that SCFAs not only modulate inflammatory responses, but also play a protective role in acute kidney injury caused by ischemia-reperfusion. Overall, SCFAs play a certain protective role in both acute and chronic kidney disease (CKD).

Our results revealed that gut bacteria were abundant in IgAN, including *Faecalibacterium*, *Megamonas*, *Prevotella*, *Escherichia-Shigella*, and *Eubacterium-coprostanoligenes*. In the intestine, LPS is a major component of the outer membrane of gram-negative bacteria including *Faecalibacterium*, *Prevotella*, *Megamonas*, and *Eubacterium-coprostanoligenes* (Wexler, 2007; Zhan et al., 2018; Hetemäki et al., 2021); it triggers systemic inflammation and releases pro-inflammatory cytokines into the systemic circulation (Cani et al., 2007; Darnaud et al., 2013; Rajilić-Stojanović and de Vos, 2014). LPS is a product of intestinal dysbiosis and overgrowth of urea-producing bacteria, which is a harmful metabolite. Data has suggested a new hypothesis that LPS may stimulate an abnormal response of tonsillar lymphocytes to alimentary antigens or commensal microbes, resulting in the synthesis of aberrantly glycosylated polymeric IgA1, which eventually enters the circulation and forms renal IgA deposits (He et al., 2013; Coppo, 2015). *Megamonas* ferments various carbohydrates; the final products are acetate, propionate, and lactate, which also interact with systemic inflammatory cytokines (Ling et al., 2016). The *Escherichia-Shigella* genus can produce amyloid and endotoxins, which cause infection (O'Callaghan and van Sinderen, 2016). The *Bifidobacterium* genus is generally considered to consist of probiotics; an abundance correlates with healthy phenotypes. The present study came to inconsistent conclusions (De Angelis et al., 2014) as to whether the abundance of *Bifidobacterium* was elevated in IgAN and n_IgAN compared with HCs. Previous studies have shown that growth of *Bifidobacteria* is affected by gender, age, and dietary habits (Mueller et al., 2006; Goetze et al., 2008),. which may be part of the reason why our study results are inconsistent with other studies.

We further observed that screened OTUs contributed greatly to establish the non-invasive classification models for IgAN. Among the top four OTUs that contributed the most to the establishment of the classification model of IgAN and HCs, two belonged to the *Lachnospira* family. The top five OTUs that contributed most to the classification model of IgAN and n_IgAN, included *Lachnospira*, *Phascolarctobacterium*, *Eubacterium-hallii*, all of which are beneficial flora, suggesting that a reduction in beneficial bacteria as a reference for model construction can achieve higher accuracy. Muscle-type phospholipase A2 receptor (PLA2R) and thrombospondintype-1 domain-containing 7A (THSD7A) were identified as the target antigens of MN. PLA2R-negative MN and other types of kidney diseases can only be differentiated from IgAN by renal biopsy. Our study offers a novel option for the non-invasive differential diagnosis of IgAN.

The gut microbiota can affect the human body by causing changes in the host metabolome or co-metabolism of substrates. Metabolites, such as tryptophan, were down-regulated, whereas others, such as beta-alanine, were up-regulated in HCs. In our study, the digestion and absorption of proteins, metabolism of amino acids (such as tryptophan, arginine, proline, tyrosine), the mechanistic target of rapamycin (mTOR) signaling pathway, aminoacyl biosynthesis, etc., were involved in IgAN. Gut-derived uremic toxins include the well-known indoxyl sulfate (IS), p-cresyl sulfate (PCS), and trimethylamine N-oxide (TMAO) (Aronov et al., 2011). Studies have shown that IS and PCS can predict the progression of CKD and are associated with poor outcomes (Hu et al., 2018). Metabolism of tryptophan can promote the production of indoles (Castillo-Rodriguez et al., 2018). In addition, enhanced metabolism of phenylalanine contributes to the production of p-cresol (Cao et al., 2015). This study found that valine, a branched chain amino acid (BCAA), was enriched in IgAN. BCAAs can activate the mTOR complex 1(mTORC1) pathway (Langenberg and Savage, 2011), which is activated in human skeletal muscle (Tremblay et al., 2007). The former is involved in many anabolic processes, including protein synthesis, cell growth, etc.

In conclusion, our study demonstrated that the gut microbiota of IgAN patients was different from that of n_IgAN patients and HCs. We further identified differential OTUs to establish classification models for the differential diagnosis of IgAN. Serum metabolites and metabolic pathways were also different in IgAN patients in comparison to HCs. We further explored the relationship among gut flora, metabolites, and clinical phenotypes, and found a variety of IgAN-related metabolites. This study provides a new insight into the association between IgAN and the gut microbiome.

## Data availability statement

The data presented in the study are deposited in the National Center for Biotechnology Information (https://www.ncbi.nlm.nih.gov/) SRA repository, accession number PRJNA752445, PRJNA801894, PRJNA832071 and PRJNA866322.

## Author contributions

ZZ and JS designed this study. JC and YD and JC collected, analyzed and interpreted the data. YD and JC drafted the manuscript. ZW, YZ, JS and ZZ modified and revised the manuscript critically for intellectual content. All authors contributed to the article and approved the submitted version.

## Funding

This work was supported by the National Natural Science Foundation of China (Grant Nos. 81873611, Recipient: ZZ), the National Natural Science Foundation of China (Grant Nos.82170738, Recipient: JS).

## Acknowledgments

We are grateful to all patients and healthy individuals who participated in this study. We thank all the authors that participated in the research. Likewise, the support given by the biobank of the First Affiliated Hospital of Zhengzhou University was also worthy of recognition.

## Conflict of interest

The authors declare that the research was conducted in the absence of any commercial or financial relationships that could be construed as a potential conflict of interest.

## Publisher’s note

All claims expressed in this article are solely those of the authors and do not necessarily represent those of their affiliated organizations, or those of the publisher, the editors and the reviewers. Any product that may be evaluated in this article, or claim that may be made by its manufacturer, is not guaranteed or endorsed by the publisher.

## Supplementary material

Supplementary Figure 1 Differential flora and KEGG pathways. **(A)**: Shannon index were decreased in IgAN and n_IgAN groups. **(B)**: At the phylum level, there were significant differences in the content of gut microbiota. KEGG pathways with differences at level 1 **(C)** and level 2 **(D)**. KEGG, Kyoto encyclopedia of genes and genomes.

Supplementary Figure 2 Phylogenetic profiles of gut microbes. **(A)**: Shannon index were decreased in IgAN. **(B)**: PCA based on distribution of OTUs of HC and IgAN. **(C)**: Comparison of microbial composition on phylum level. **(D)**: Comparison of differential microorganisms on phylum level. Log_10_ abundance of differential microorganisms on genus level (**P*<0.05; ***P*<0.01; ****P*<0.001). **(E)**: Based on the LDA value distribution histogram in LefSe analysis results (with the absolute value of LDA value greater than 3 as the default value), 11 genera were significantly enriched in IgAN, while 13 genera were significantly enriched in HC. The POD value was significantly increased in IgAN versus HC in the training phase **(F)**, test phase **(G)** and independent test phase **(H)**. PCA, Principal Components Analysis; LDA, Liner Discriminant Analysis; LefSe, the LDA Effect Size; POD, probability of disease.

Supplementary Figure 3 Correlation of intestinal microflora, differential metabolites and clinical phenotypes and differential metabolites. **(A)**: Different metabolites and clinical indicators. **(B)**: The correlation analysis of different flora and clinical phenotypes (**P*<0.05; ***P*<0.01; ****P*<0.001). Plasma metabolomics in the OPLS-DA model. There are obviously different distribution trends in the positive ion mode **(C)** and the negative ion mode **(D)**. **(E and F)**: Five differential metabolites whose expression were up-regulated in IgAN, and five differential metabolites whose expression were down-regulated in IgAN. OPLS-DA, Orthogonal Partial Least Squares-Discriminant Analysis; HB, hemoglobin; BUN, blood urea nitrogen; Scr, Serum creatinine; UA, uric acid; ALB, serum albumin; T-CHO, total cholesterol; TG, triglycerides; HDL, high density lipoprotein; LDL, low density lipoprotein; eGFR, estimated glomerular filtration rate; 24hTP, 24-hour urinary total protein levels; HC, healthy controls; IgAN, immunoglobulin A nephropathy.

Supplementary Figure 4 Phylogenetic profiles of gut microbes. **(A)**: Shannon index were increased in IgAN. **(B)**: PCA based on the distribution of intestinal microbe OTUs of IgAN and n_IgAN. **(C)**: Comparison of microbial composition on phylum level. **(D)**: Comparison of differential microorganisms on phylum level. Log_10_ abundance of differential microorganisms on genus level (**P*<0.05; ***P*<0.01; ****P*<0.001). **(E)**: According to the LDA value distribution histogram, 24 genera were significantly enriched in n_IgAN, while 21 genera were significantly enriched in IgAN. The POD value was significantly decreased in IgAN versus n_IgAN in the training phase **(F)**, test phase **(G)**. PCA, Principal Components Analysis; LDA, Liner Discriminant Analysis; LEfSe, the LDA Effect Size; POD, probability of disease.

Supplementary Table 1 Comparison of gut microbiota richness and evenness among three groups. Used the Mann-Whitney U test for pairwise comparisons of alpha diversity among three groups, the results were presented using the median [IQR]. IQR, interquartile range; HC, healthy controls; IgAN, immunoglobulin A nephropathy; n_IgAN, non-immunoglobulin A nephropathy.

## References

[B1] Andrade-Oliveira V. Amano M. Correa-Costa M. Castoldi A. Felizardo R. de Almeida D.. (2015). Gut bacteria products prevent AKI induced by ischemia-reperfusion. J. Am. Soc. Nephrol. JASN 26 (8), 1877–1888. doi: 10.1681/asn.2014030288 25589612 PMC4520159

[B2] Aronov P. Luo F. Plummer N. Quan Z. Holmes S. Hostetter T.. (2011). Colonic contribution to uremic solutes. J. Am. Soc. Nephrol. JASN 22 (9), 1769–1776. doi: 10.1681/asn.2010121220 21784895 PMC3171947

[B3] Béné M. Faure G. (1988). Mesangial IgA in IgA nephropathy arises from the mucosa. Am. J. Kidney Dis. 12 (5), 406–409. doi: 10.1016/s0272-6386(88)80035-0 3055966

[B4] Berger J. Hinglais N. (1968). [Intercapillary deposits of IgA-IgG]. J. d'urol. Nephrol. 74 (9), 694–695. 4180586

[B5] Cani P. Amar J. Iglesias M. Poggi M. Knauf C. Bastelica D.. (2007). Metabolic endotoxemia initiates obesity and insulin resistance. Diabetes 56 (7), 1761–1772. doi: 10.2337/db06-1491 17456850

[B6] Cao X. Chen J. Zou J. Zhong Y. Teng J. Ji J.. (2015). Association of indoxyl sulfate with heart failure among patients on hemodialysis. Clin. J. Am. Soc. Nephrol. CJASN 10 (1), 111–119. doi: 10.2215/cjn.04730514 25332316 PMC4284412

[B7] Castillo-Rodriguez E. Fernandez-Prado R. Esteras R. Perez-Gomez M. Gracia-Iguacel C. Fernandez-Fernandez B.. (2018). Impact of altered intestinal microbiota on chronic kidney disease progression. Toxins 10 (7), 1–21. doi: 10.3390/toxins10070300 PMC6070989 30029499

[B8] Chou Y. Lien Y. Hu F. Lin W. Kao C. Lai C.. (2012). Clinical outcomes and predictors for ESRD and mortality in primary GN. Clin. J. Am. Soc. Nephrol. CJASN 7 (9), 1401–1408. doi: 10.2215/cjn.04500511 22798538 PMC3430959

[B9] Coppo R. (2015). The intestine-renal connection in IgA nephropathy. Nephrol. Dial. Transplant. 30 (3), 360–366. doi: 10.1093/ndt/gfu343 25387475

[B10] Coppo R. (2018). The gut-renal connection in IgA nephropathy. Semin. Nephrol. 38 (5), 504–512. doi: 10.1016/j.semnephrol.2018.05.020 30177022

[B11] Darnaud M. Faivre J. Moniaux N. (2013). Targeting gut flora to prevent progression of hepatocellular carcinoma. J. Hepatol. 58 (2), 385–387. doi: 10.1016/j.jhep.2012.08.019 22940407

[B12] De Angelis M. Montemurno E. Piccolo M. Vannini L. Lauriero G. Maranzano V.. (2014). Microbiota and metabolome associated with immunoglobulin a nephropathy (IgAN). PloS One 9 (6), e99006. doi: 10.1371/journal.pone.0099006 24922509 PMC4055632

[B13] DeSoignie R. Sellin J. (1994). Propionate-initiated changes in intracellular pH in rabbit colonocytes. Gastroenterology 107 (2), 347–356. doi: 10.1016/0016-5085(94)90158-9 8039611

[B14] Dong R. Bai M. Zhao J. Wang D. Ning X. Sun S. (2020). A comparative study of the gut microbiota associated with immunoglobulin a nephropathy and membranous nephropathy. Front. Cell. Infect. Microbiol. 10. doi: 10.3389/fcimb.2020.557368 PMC7606180 33194798

[B15] Gesualdo L. Di Leo V. Coppo R. (2021). The mucosal immune system and IgA nephropathy. Semin. Immunopathol. 43 (5), 657–668. doi: 10.1007/s00281-021-00871-y 34642783 PMC8551125

[B16] Goetze O. Fruehauf H. Pohl D. Giarrè M. Rochat F. Ornstein K.. (2008). Effect of a prebiotic mixture on intestinal comfort and general wellbeing in health. Br. J. Nutr. 100 (5), 1077–1085. doi: 10.1017/s0007114508960918 18377682

[B17] He L. Peng Y. Liu H. Yin W. Chen X. Peng X.. (2013). Activation of the interleukin-4/signal transducer and activator of transcription 6 signaling pathway and homeodomain-interacting protein kinase 2 production by tonsillar mononuclear cells in IgA nephropathy. Am. J. Nephrol. 38 (4), 321–332. doi: 10.1159/000355393 24107646

[B18] Hetemäki I. Jian C. Laakso S. Mäkitie O. Pajari A. de Vos W.. (2021). Fecal bacteria implicated in biofilm production are enriched and associate to gastrointestinal symptoms in patients with APECED - a pilot study. Front. Immunol. 12. doi: 10.3389/fimmu.2021.668219 PMC8339580 34367134

[B19] Hu J. Coresh J. Inker L. Levey A. Zheng Z. Rebholz C.. (2018). Serum metabolites are associated with all-cause mortality in chronic kidney disease. Kidney Int. 94 (2), 381–389. doi: 10.1016/j.kint.2018.03.008 29871777 PMC6054894

[B20] Hu R. Quan S. Wang Y. Zhou Y. Zhang Y. Liu L.. (2020). Spectrum of biopsy proven renal diseases in central China: a 10-year retrospective study based on 34,630 cases. Sci. Rep. 10 (1), 10994. doi: 10.1038/s41598-020-67910-w 32620914 PMC7335090

[B21] Kano T. Suzuki H. Makita Y. Fukao Y. Suzuki Y. (2021). Nasal-associated lymphoid tissue is the major induction site for nephritogenic IgA in murine IgA nephropathy. Kidney Int. 100 (2), 364–376. doi: 10.1016/j.kint.2021.04.026 33961870

[B22] Lai K. Tang S. Schena F. Novak J. Tomino Y. Fogo A.. (2016). IgA nephropathy. Nat. Rev. Dis. Primers 2, 16001. doi: 10.1038/nrdp.2016.1 27189177

[B23] Langenberg C. Savage D. (2011). An amino acid profile to predict diabetes? Nat. Med. 17 (4), 418–420. doi: 10.1038/nm0411-418 21475231

[B24] Ling Z. Jin C. Xie T. Cheng Y. Li L. Wu N. (2016). Alterations in the fecal microbiota of patients with HIV-1 infection: An observational study in a Chinese population. Sci. Rep. 6, 30673. doi: 10.1038/srep30673 27477587 PMC4967929

[B25] Makita Y. Suzuki H. Kano T. Takahata A. Julian B. Novak J.. (2020). TLR9 activation induces aberrant IgA glycosylation *via* APRIL- and IL-6-mediated pathways in IgA nephropathy. Kidney Int. 97 (2), 340–349. doi: 10.1016/j.kint.2019.08.022 31748116 PMC7372907

[B26] Mueller S. Saunier K. Hanisch C. Norin E. Alm L. Midtvedt T.. (2006). Differences in fecal microbiota in different European study populations in relation to age, gender, and country: a cross-sectional study. Appl. Environ. Microbiol. 72 (2), 1027–1033. doi: 10.1128/aem.72.2.1027-1033.2006 16461645 PMC1392899

[B27] O'Callaghan A. van Sinderen D. (2016). Bifidobacteria and their role as members of the human gut microbiota. Front. Microbiol. 7. doi: 10.3389/fmicb.2016.00925 PMC4908950 27379055

[B28] O'Shaughnessy M. Hogan S. Poulton C. Falk R. Singh H. Nickeleit V.. (2017). Temporal and demographic trends in glomerular disease epidemiology in the southeastern united states 1986-2015. Clin. J. Am. Soc. Nephrol. CJASN 12 (4), 614–623. doi: 10.2215/cjn.10871016 28325866 PMC5383393

[B29] Qu L. Dong Z. Ma S. Liu Y. Zhou W. Wang Z.. (2022). Gut microbiome signatures are predictive of cognitive impairment in hypertension patients-a cohort study. Front. Microbiol. 13. doi: 10.3389/fmicb.2022.841614 PMC9024414 35464979

[B30] Rajilić-Stojanović M. de Vos W. (2014). The first 1000 cultured species of the human gastrointestinal microbiota. FEMS Microbiol. Rev. 38 (5), 996–1047. doi: 10.1111/1574-6976.12075 24861948 PMC4262072

[B31] Sabatino A. Regolisti G. Cosola C. Gesualdo L. Fiaccadori E. (2017). Intestinal microbiota in type 2 diabetes and chronic kidney disease. Curr. Diabetes Rep. 17 (3), 16. doi: 10.1007/s11892-017-0841-z 28271466

[B32] Sallustio F. Curci C. Chaoul N. Fontò G. Lauriero G. Picerno A.. (2021). High levels of gut-homing immunoglobulin a+ b lymphocytes support the pathogenic role of intestinal mucosal hyperresponsiveness in immunoglobulin a nephropathy patients. Nephrol. Dial. Transplant. 36 (3), 452–464. doi: 10.1093/ndt/gfaa264 33200215 PMC7898021

[B33] Shin N. Whon T. Bae J. (2015). Proteobacteria: microbial signature of dysbiosis in gut microbiota. Trends Biotechnol. 33 (9), 496–503. doi: 10.1016/j.tibtech.2015.06.011 26210164

[B34] Sugurmar A. Mohd R. Shah S. Neoh H. Cader R. (2021). Gut microbiota in immunoglobulin a nephropathy: a Malaysian perspective. BMC Nephrol. 22 (1), 145. doi: 10.1186/s12882-021-02315-z 33882859 PMC8060124

[B35] Suzuki H. Fan R. Zhang Z. Brown R. Hall S. Julian B.. (2009). Aberrantly glycosylated IgA1 in IgA nephropathy patients is recognized by IgG antibodies with restricted heterogeneity. J. Clin. Invest. 119 (6), 1668–1677. doi: 10.1172/jci38468 19478457 PMC2689118

[B36] Suzuki H. Yasutake J. Makita Y. Tanbo Y. Yamasaki K. Sofue T.. (2018). IgA nephropathy and IgA vasculitis with nephritis have a shared feature involving galactose-deficient IgA1-oriented pathogenesis. Kidney Int. 93 (3), 700–705. doi: 10.1016/j.kint.2017.10.019 29329643

[B37] Tan J. McKenzie C. Potamitis M. Thorburn A. Mackay C. Macia L. (2014). The role of short-chain fatty acids in health and disease. Adv. Immunol. 121, 91–119. doi: 10.1016/b978-0-12-800100-4.00003-9 24388214

[B38] Tremblay F. Brûlé S. Hee Um S. Li Y. Masuda K. Roden M.. (2007). Identification of IRS-1 ser-1101 as a target of S6K1 in nutrient- and obesity-induced insulin resistance. Proc. Natl. Acad. Sci. United States America 104 (35), 14056–14061. doi: 10.1073/pnas.0706517104 PMC1950339 17709744

[B39] Verkaik N. Boelens H. de Vogel C. Tavakol M. Bode L. Verbrugh H.. (2010). Heterogeneity of the humoral immune response following staphylococcus aureus bacteremia. Eur. J. Clin. Microbiol. Infect. Dis. 29 (5), 509–518. doi: 10.1007/s10096-010-0888-0 20186449 PMC2854366

[B40] Vicentini F. Keenan C. Wallace L. Woods C. Cavin J. Flockton A.. (2021). Intestinal microbiota shapes gut physiology and regulates enteric neurons and glia. Microbiome 9 (1), 210. doi: 10.1186/s40168-021-01165-z 34702353 PMC8549243

[B41] Wang S. Lv D. Jiang S. Jiang J. Liang M. Hou F.. (2019). Quantitative reduction in short-chain fatty acids, especially butyrate, contributes to the progression of chronic kidney disease. Clin. Sci. (London Engl. 1979) 133 (17), 1857–1870. doi: 10.1042/cs20190171 31467135

[B42] Wexler H. (2007). Bacteroides: the good, the bad, and the nitty-gritty. Clin. Microbiol. Rev. 20 (4), 593–621. doi: 10.1128/cmr.00008-07 17934076 PMC2176045

[B43] Wong J. Piceno Y. DeSantis T. Pahl M. Andersen G. Vaziri N. (2014). Expansion of urease- and uricase-containing, indole- and p-cresol-forming and contraction of short-chain fatty acid-producing intestinal microbiota in ESRD. Am. J. Nephrol. 39 (3), 230–237. doi: 10.1159/000360010 24643131 PMC4049264

[B44] Wyatt R. Julian B. (2013). IgA nephropathy. New Engl. J. Med. 368 (25), 2402–2414. doi: 10.1056/NEJMra1206793 23782179

[B45] Zhan X. Stamova B. Sharp F. (2018). Lipopolysaccharide associates with amyloid plaques, neurons and oligodendrocytes in alzheimer's disease brain: A review. Front. Aging Neurosci. 10. doi: 10.3389/fnagi.2018.00042 PMC5827158 29520228

[B46] Zhong Z. Tan J. Tan L. Tang Y. Qiu Z. Pei G.. (2020). Modifications of gut microbiota are associated with the severity of ig a nephropathy in the Chinese population. Int. Immunopharmacol. 89 (Pt B), 107085. doi: 10.1016/j.intimp.2020.107085 33068859

