# Supplementary figures and images for: Development and validation of diagnostic models for immunoglobulin A nephropathy based on gut microbes
Source: Front Cell Infect Microbiol. 2022 Dec 8;12:1059692. doi: 10.3389/fcimb.2022.1059692 (PMC9774022; doi:10.3389/fcimb.2022.1059692)

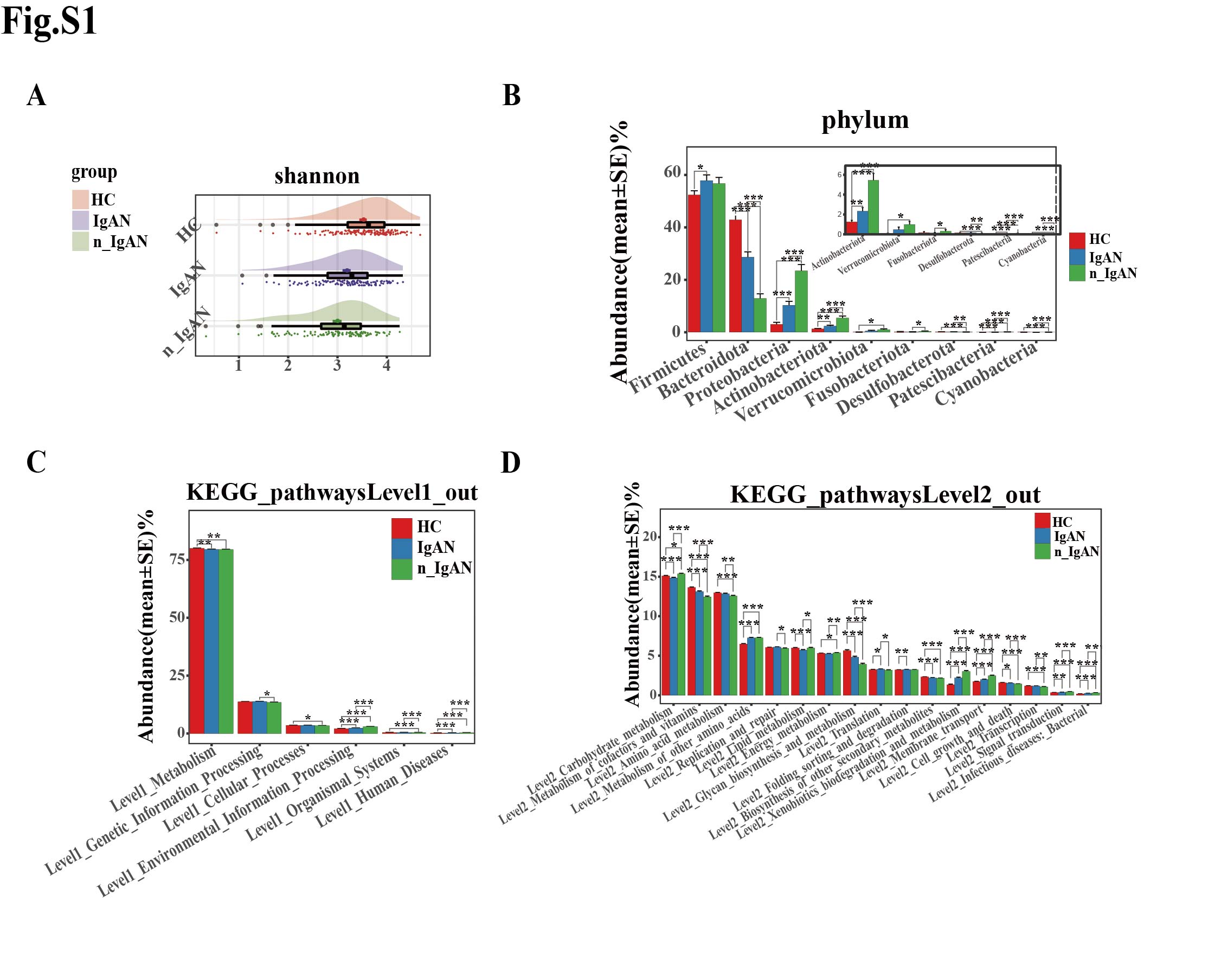

Supplement: Supplementary Figure 1 — Differential flora and KEGG pathways. (A): Shannon index were decreased in IgAN and n_IgAN groups. (B): At the phylum level, there were significant differences in the content of gut microbiota. KEGG pathways with differences at level 1 (C) and level 2 (D). KEGG, Kyoto encyclopedia of genes and genomes. [file Image_1.jpg]

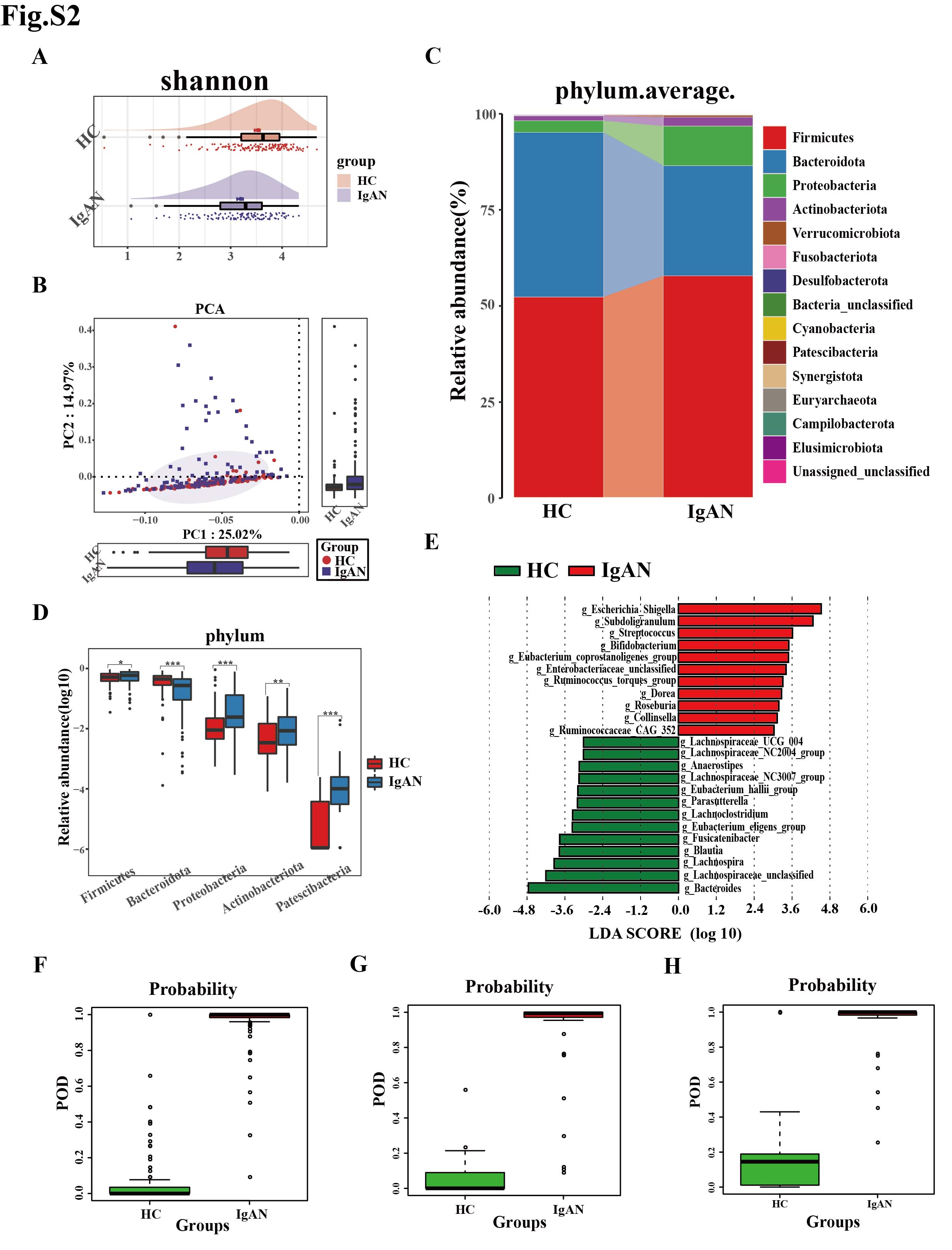

Supplement: Supplementary Figure 2 — Phylogenetic profiles of gut microbes. (A): Shannon index were decreased in IgAN. (B): PCA based on distribution of OTUs of HC and IgAN. (C): Comparison of microbial composition on phylum level. (D): Comparison of differential microorganisms on phylum level. Log10 abundance of differential microorganisms on genus level (*P<0.05; **P<0.01; ***P<0.001). (E): Based on the LDA value distribution histogram in LefSe analysis results (with the absolute value of LDA value greater than 3 as the default value), 11 genera were significantly enriched in IgAN, while 13 genera were significantly enriched in HC. The POD value was significantly increased in IgAN versus HC in the training phase (F), test phase (G) and independent test phase (H). PCA, Principal Components Analysis; LDA, Liner Discriminant Analysis; LefSe, the LDA Effect Size; POD, probability of disease. [file Image_2.jpg]

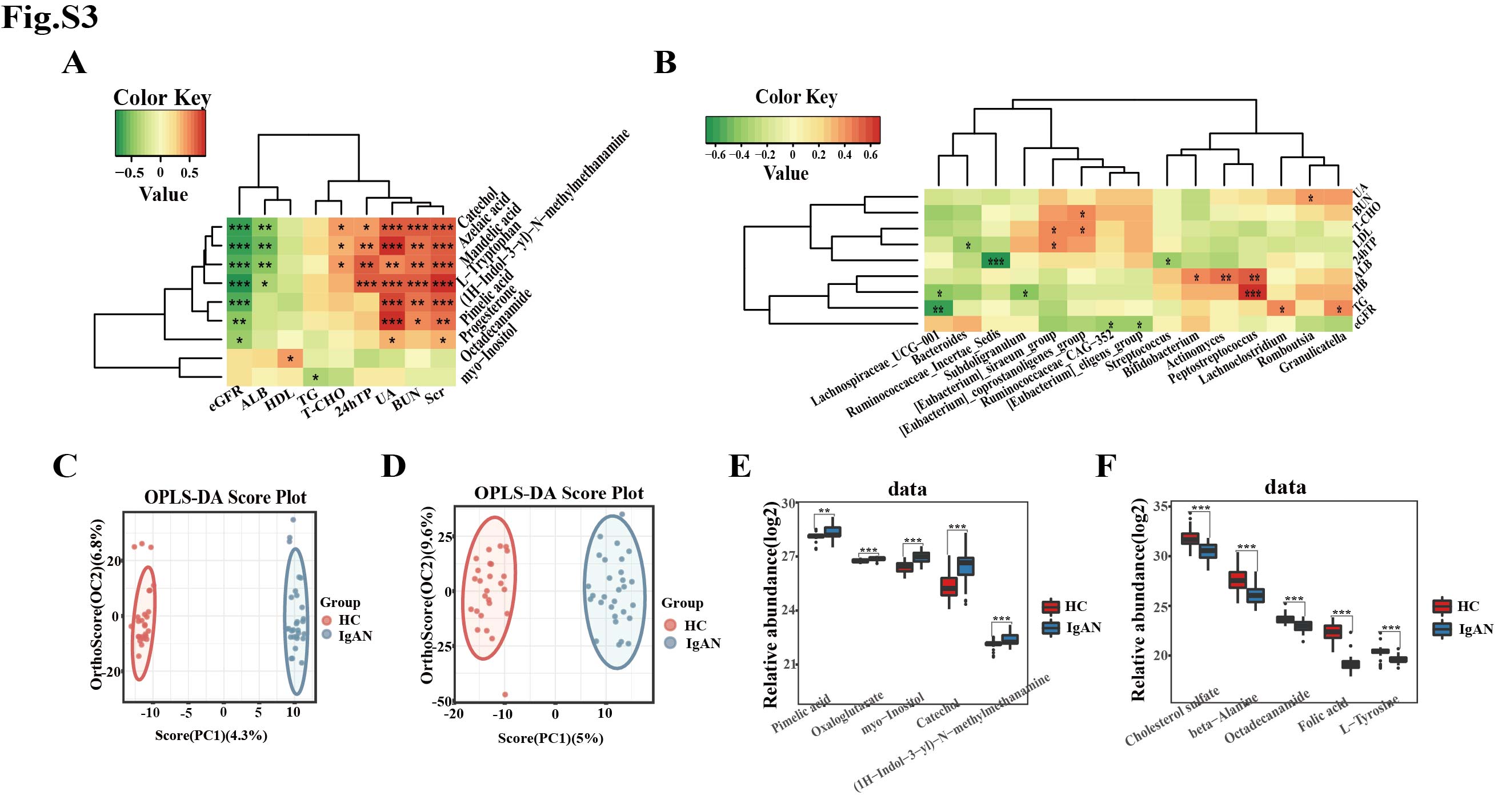

Supplement: Supplementary Figure 3 — Correlation of intestinal microflora, differential metabolites and clinical phenotypes and differential metabolites. (A): Different metabolites and clinical indicators. (B): The correlation analysis of different flora and clinical phenotypes (*P<0.05; **P<0.01; ***P<0.001). Plasma metabolomics in the OPLS-DA model. There are obviously different distribution trends in the positive ion mode (C) and the negative ion mode (D). (E and F): Five differential metabolites whose expression were up-regulated in IgAN, and five differential metabolites whose expression were down-regulated in IgAN. OPLS-DA, Orthogonal Partial Least Squares-Discriminant Analysis; HB, hemoglobin; BUN, blood urea nitrogen; Scr, Serum creatinine; UA, uric acid; ALB, serum albumin; T-CHO, total cholesterol; TG, triglycerides; HDL, high density lipoprotein; LDL, low density lipoprotein; eGFR, estimated glomerular filtration rate; 24hTP, 24-hour urinary total protein levels; HC, healthy controls; IgAN, immunoglobulin A nephropathy. [file Image_3.jpg]

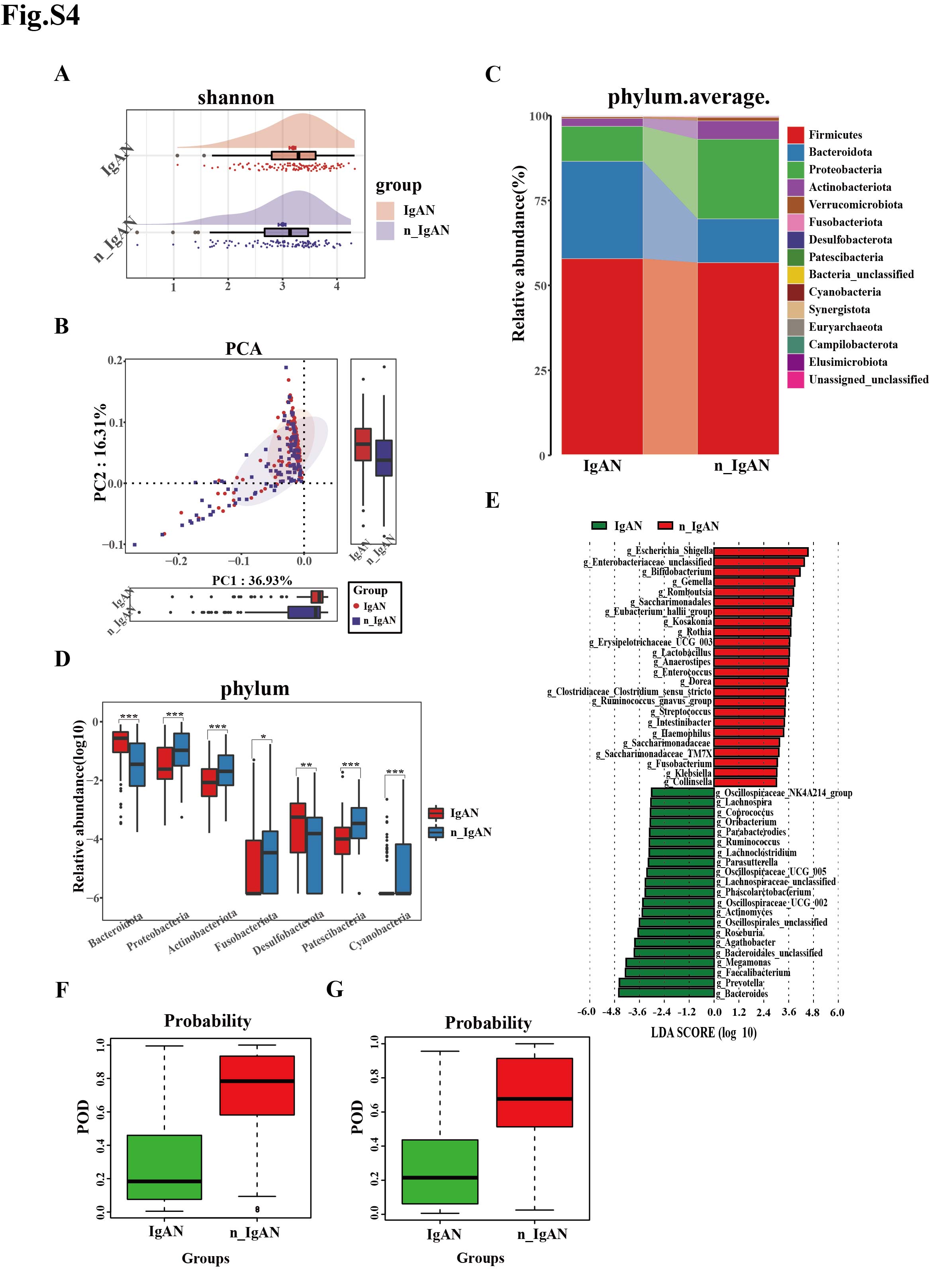

Supplement: Supplementary Figure 4 — Phylogenetic profiles of gut microbes. (A): Shannon index were increased in IgAN. (B): PCA based on the distribution of intestinal microbe OTUs of IgAN and n_IgAN. (C): Comparison of microbial composition on phylum level. (D): Comparison of differential microorganisms on phylum level. Log10 abundance of differential microorganisms on genus level (*P<0.05; **P<0.01; ***P<0.001). (E): According to the LDA value distribution histogram, 24 genera were significantly enriched in n_IgAN, while 21 genera were significantly enriched in IgAN. The POD value was significantly decreased in IgAN versus n_IgAN in the training phase (F), test phase (G). PCA, Principal Components Analysis; LDA, Liner Discriminant Analysis; LEfSe, the LDA Effect Size; POD, probability of disease. [file Image_4.jpg]
