# Supplementary material for: Development and validation of diagnostic models for immunoglobulin A nephropathy based on gut microbes
Source: Front Cell Infect Microbiol. 2022 Dec 8;12:1059692. doi: 10.3389/fcimb.2022.1059692 (PMC9774022; doi:10.3389/fcimb.2022.1059692)
Supplement: Supplementary Table 1 — Comparison of gut microbiota richness and evenness among three groups. Used the Mann-Whitney U test for pairwise comparisons of alpha diversity among three groups, the results were presented using the median [IQR]. IQR, interquartile range; HC, healthy controls; IgAN, immunoglobulin A nephropathy; n_IgAN, non-immunoglobulin A nephropathy. [file Table_1.docx]

**Table S1:** **Comparison of gut microbiota richness and evenness among three groups.** Used the Mann-Whitney U test for pairwise comparisons of alpha diversity among three groups, the results were presented using the median [IQR]. IQR, interquartile range; HC, healthy controls; IgAN, immunoglobulin A nephropathy; n_IgAN, non-immunoglobulin A nephropathy.

| Alpha_diversity  (median[IQR]) | HC-median | IgAN-median | n_IgAN-median | p-value |
| --- | --- | --- | --- | --- |
| ace | 540.8337[419.9889,635.7195] | 430.7192[323.6208,527.7354] | 360.705[284.2339,431.3003] | P<0.001^a,b,c^ |
| chao | 539.1193[415.9832,640.1591] | 418.8182[331.5,518.7179] | 350.4435[259.1005,421.2038] | P<0.001^a,b,c^ |
| shannon | 3.6284[3.2049,3.9487] | 3.2912[2.799,3.6073] | 3.137[2.669,3.4699] | P<0.001^a,b^ |
| simpson | 0.076[0.0491,0.1168] | 0.0968[0.0694,0.1454] | 0.0963[0.0647,0.1893] | P<0.01^a,b^ |
| observed_otus | 417[325,492.5] | 321[241,395] | 259[194,311.25] | P<0.001^a,b,c^ |

| a: HC: IgAN |
| --- |
| b: HC: n_IgAN |
| c: IgAN: n_IgAN |
